# Intradural schwannoma complicated by lumbar disc herniation at the same level: A case report and review of the literature

**DOI:** 10.3892/ol.2014.2181

**Published:** 2014-05-26

**Authors:** SEUNG-WOOK BAEK, CHEOL KIM, HAN CHANG

**Affiliations:** Department of Orthopaedic Surgery, Spine Center, Busan Korea Hospital, Busan 608-811, Republic of Korea

**Keywords:** herniated intervertebral disc, intradural tumor, schwannoma

## Abstract

Intradural tumours of the spine are usually benign and have a good prognosis, if they are diagnosed and removed early. Lumbar disc herniation is a common cause of chronic, acute, or recurrent lumbar radiculopathy. However, to date, there have been no reports of progressive neurological deficiencies due to the co-existence of two significant pathologies contributing to intradural and extradural compression. The current study reports the rare case of a patient with simultaneous extradural and intradural compression of the nerve root due to co-existent intervertebral disc herniation and an intradural schwannoma at the same level. A 71-year-old female suffering from lower back pain and radiating pain of the right lower extremities was admitted to Busan Korea Hospital (Busan, Korea). Magnetic resonance imaging revealed lumbar disc herniation at L4–5 and a mass occupying the intradural space at the same level of the compressed dural sac. Using the posterior approach, surgical excision of the two pathologies was performed. Pathological diagnosis confirmed schwannoma and the symptoms markedly improved.

## Introduction

Lumbar disc herniation is the most common cause of lumbar radiculopathy (1). The majority of patients with lumbar disc herniation respond to conservative treatment, but for those with persistent or progressive symptoms of nerve root compression, surgical treatment must be considered (2,3). Schwannomas are slow-growing, benign, encapsulated tumors that originate from the Schwann cells in the myelin sheath of nerve fibers (4). Schwannomas are generally single tumors and account for 26% of all intraspinal tumors that involve the lumbar spinal nerves. In addition, they are usually benign, but can be locally aggressive and cause neurological compromise (5). At present, no studies have reported the co-existence of these pathologies and thus may confuse physicians. This report presents a rare case of the co-existence of two significant pathologies contributing to intradural and extradural compression. The patient provided written informed consent.

## Case report

A 75-year-old female had been experiencing lower back and right lower extremity pain since May 2011, which led them to seek help from local medical clinics. The patient’s symptoms were occasionally alleviated by medication and physiotherapy.

**Figure 1:**
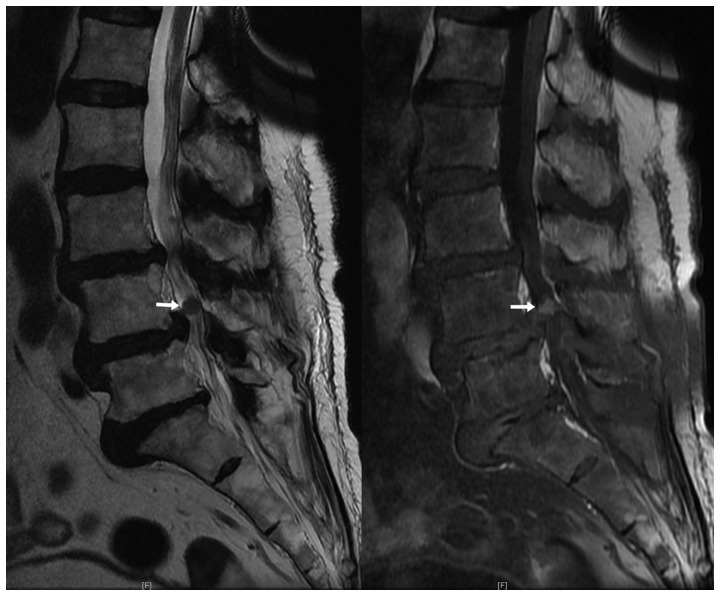
T2-weighted (left) and enhanced T1-weighted (right) magnetic resonance imaging revealing a well-rounded enhanced mass at the level of the L4–5 disc space, as shown by the white arrows.

In January 2013, the patient visited an orthopedist. Plain films of the lumbar spine revealed lumbar spondylosis, which did not improve with pharmacotherapy. The patient then underwent a magnetic resonance imaging (MRI) scan of the lumbar region, confirming the diagnosis of spinal stenosis and intradural tumor. An incidental intradural structure, which following gadolinium injection became increasingly distinct, was noted and diagnosed as a schwannoma (Fig. 1). The patient then visited the Department of Orthopaedic Surgery, Busan Korea Hospital (Busan, Korea) to be evaluated for surgical management. As the patient exhibited improvement with medication and physiotherapy, it was recommended that conservative treatment would remain as the first-line treatment and surgery would only be considered when the former was no longer effective.

In May 2013, the patient’s lower back pain worsened suddenly and the patient experienced numbness and motor weakness of the right lower extremities. Neurological examination revealed weakness of the extensor hallucis longus and the gluteus medius. The patient reported abnormal sensations in the right buttock and lateral leg, however, bladder and bowel function were normal, and no reduction in deep tendon reflex was identified. The results of the straight leg-raising test were 40°/70° and the Patrick’s test results were normal. These observations led to a suspected diagnosis of a rapidly growing tumor with right L5 radiculopathy.

**Figure 2:**
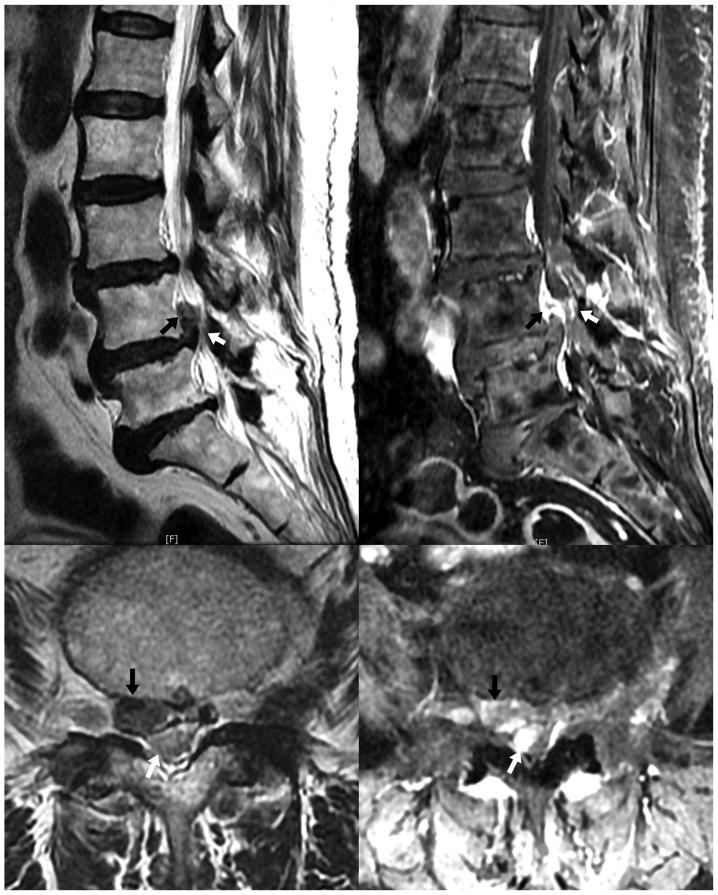
T2-weighted (left) and enhanced T1-weighted (right) sagittal (top) and axial (bottom) magnetic resonance imaging revealing disc herniation compressing the thecal sac and right neural foramen at L4/5, shown by the black arrows, and an intradural structure of increased density following enhancement at the same site, shown by the white arrows.

However, follow-up MRI scan revealed lumbar disc herniation compressing the thecal sac and right neural foramen at L4/5, and an intradural structure at the same level (Fig. 2). To remove the mass, the patient underwent total L4 laminectomy. Microscopically, the intradural tumor was successfully removed. The dura mater of the L4/5 was opened microsurgically, allowing the nerve fibers involved in the tumor to be identified. The involved fibers surrounding the tumor were cut, and the lesion was resected, preserving the intact nerve fibers. An upward migrated lumbar disc herniation at L4/5 was also successfully removed.

**Figure 3:**
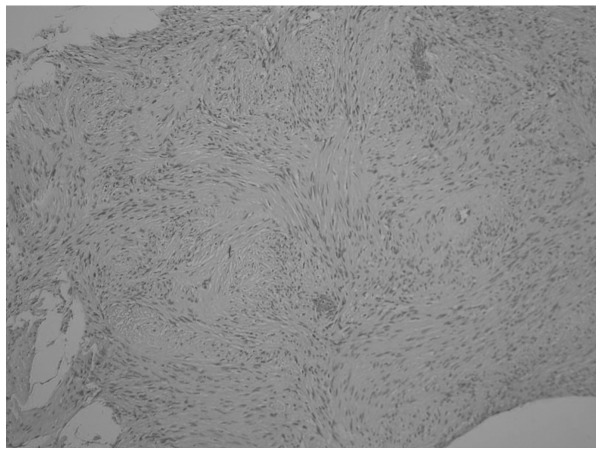
Pathological analysis shows compact spindled areas composed of cells with elongated and tightly packed nuclei (stain, hematoxylin and eosin; magnification, ×200).

The pathological report confirmed the tumor to be a schwannoma (Fig. 3). The neurological status improved by the sixth postoperative week. At the postoperative eighth month, the patient’s symptoms improved significantly, with only a residual abnormal sensation on the skin of the left buttock, which did not affect the patient’s normal active life style.

## Discussion

The present case report describes a patient with intradural schwannoma whose symptoms initially improved with conservative treatment, but later worsened with neurological deficit. Further investigation using contrast-enhanced MRI incidentally revealed disc herniation at the site of the intradural tumor, which was exacerbating the pre-existing symptoms.

Coexistence of a lumbar disc herniation and a proximal dorsal or cervical intradural tumor has not been reported in cancer patients (6). The simultaneous presence of upper and lower motor neuron symptoms in the extremities validate the diagnosis. In the case of lumbar intradural tumors, it is not uncommon for these lesions to simulate symptoms of a prolapsed intervertebral disc (7). In the present case, the two lesions compressed the cauda equina at the same level. Therefore, the intradural tumor and herniated intervertebral discs were removed.

The majority of intradural tumors follow a slow progressive, indolent course and, thus, there may be lag period of several months prior to the symptoms becoming evident (8,9). Lower back pain and cauda equina syndrome generally result from larger tumors, which impinge on multiple spinal root levels (10,11), and tumors in the cauda equina often reach a considerable size without painful symptoms, due to the mobility of the roots and the wide intradural space (12). However, the neurological impairment of the patient presented in this case resulted from intradural and extradural compression. Therefore, the possibility of tumor growth or increased disc herniation must be ruled out to prevent delayed diagnosis and treatment in cases where symptoms worsen, and in patients undergoing conservative treatment for lumbar herniated intervertebral disk (HIVD). Additionally, if symptoms acutely worsen, the possibility of an intraspinal tumor must be considered.

The detection of small and slow-growing schwannoma accompanied by lumbar HIVD requires the use of MRI with contrast enhancement. Although schwannoma and herniated IVD appeared bright on T2-weighted MRI, only the schwannoma was shown by gadolinium and, thus, the use of different contrast enhancement may be used to distinguish between the two (13).

The preoperative detection of a schwannoma exhibits a significantly positive impact on the outcome of surgery and postoperative recovery. Spinal nerve compression due to tumors or HIVD cannot be easily distinguished clinically. Therefore, a complete history must be obtained and physical examination in conjunction with investigative measures, such as MRI with contrast, must be performed to ensure early diagnosis and implementation of adequate treatment. Furthermore, physicians must not confirm the diagnosis without performing full adequate studies.
